# Application of variable selection in the origin discrimination of *Wolfiporia cocos* (F.A. Wolf) Ryvarden & Gilb. based on near infrared spectroscopy

**DOI:** 10.1038/s41598-017-18458-9

**Published:** 2018-01-08

**Authors:** Tianjun Yuan, Yanli Zhao, Ji Zhang, Yuanzhong Wang

**Affiliations:** 1 Institute of Medicinal Plants, Yunnan Academy of Agricultural Sciences, Kunming, 650200 China; 2 Yunnan Comtestor CO., LTD., Kunming, 650106 China

## Abstract

Dried sclerotium of *Wolfiporia cocos* (F.A. Wolf) Ryvarden & Gilb. is a traditional Chinese medicine. Its chemical components showed difference among geographical origins, which made it difficult to keep therapeutic potency consistent. The identification of the geographical origin of *W. cocos* is the fundamental prerequisite for its worldwide recognition and acceptance. Four variable selection methods were employed for near infrared spectroscopy (NIR) variable selection and the characteristic variables were screened for the establishment of Fisher function models in further identification of the origin of *W. cocos* from Yunnan, China. For the obvious differences between poriae cutis (fu-ling-pi in Chinese, or FLP) and the inner part (bai-fu-ling in Chinese, or BFL) of the sclerotia of *W. cocos* in the pattern space of principal component analysis (PCA), we established discriminant models for FLP and BFL separately. Through variable selection, the models were significant improved and also the models were simplified by using only a small part of the variables. The characteristic variables were screened (13 for BFL and 10 for FLP) to build Fisher discriminant function models and the validation results showed the models were reliable and effective. Additionally, the characteristic variables were interpreted.

## Introduction

Dried sclerotia of *Wolfiporia cocos* (F.A. Wolf) Ryvarden & Gilb. is a well-known traditional Chinese medicine, which is a fungal species parasitizing the roots of pine trees^1^. Traditionally, it is used in many prescriptions for inducing diuresis, invigorating the spleen, excreting dampness and tranquilizing the mind. However, poriae cutis (fu-ling-pi in Chinese, or FLP) and the inner part (bai-fu-ling in Chinese, or BFL) of the sclerotia of *W. cocos* have different therapeutic efficacy. FLP is reported to have only diuretic activity, while BFL has an invigorating activity in addition to diuretic and sedative effects^2^. Modern phytochemical and pharmacological investigations have shown that triterpenes and polysaccharides are the two main kinds of secondary metabolites found in *W. cocos*, which are responsible for its functions of anti-tumor, anti-oxidant, anti-rejection, antibacterial, anti-inflammatory, anti-hyperglycemic, nematicidal, etc^3^. The previous studies found that the contents of triterpenoid and polysaccharide in *W. cocos* from different origins were different^4,5^. The difference in chemical components of *W. cocos* in different geographical origins makes it difficult to keep therapeutic potency consistent. The identification of the geographical origin of *W. cocos* is the fundamental prerequisite for its worldwide recognition and acceptance.

In China, the poria produced in Yunnan is reputable as Yunnan poria (Yun-ling in Chinese) for its geoherbalism. Yunnan locates in southwest China and is influenced by a low latitude plateau, mountainous country monsoon climate^6^. There are seven climatic zones in Yunnan from the north temperate zone to north tropic zone, and climatic zones distribute according to the elevation^7^. The complex climate condition influences the quality of *W. cocos*. It was reported that the infrared spectra of *W. cocos* peels from different producing areas (Hubei, Anhui and Yunnan provinces) revealed obvious regional differences, and for the large geographical span, the component contents in samples from Yunnan were different at a certain extent^8^. Based on ultra performance liquid chromatography-ultraviolet-mass spectrometry (UPLC-UV-MS) fingerprints, the effect of habitat on the quality of peeled and sliced poria was obvious^9^.

Near-infrared spectroscopy (NIR), as a fast and non-destructive technology, has been widely used to identify traditional Chinese medicinal materials^10–14^. The NIR spectrum reflects the absorption of overtones and combinations of the fundamental mid-IR bands like C-H, O-H, and N-H functional groups. The bandwidth of NIR region (between 780 and 2500 nm (12000 to 4000 cm^−1^)) is wide and absorption bands overlap heavily, which make the analysis of NIR spectra extremely difficult with conventional methods^15,16^. The variable selection is a critical step in the analysis of the datasets with thousands of variables in NIR spectroscopy^17^. In recent years, several variable selection methods of NIR have been developed, such as interval partial least-squares (iPLS)^18,19^, backward interval partial least-squares (biPLS)^20^, moving window partial least-squares regression (MWPLSR)^21^, genetic algorithm (GA)^22–24^, simulated annealing algorithm (SAA)^25^, competitive adaptive reweighted sampling (CARS)^26–28^, Monte Carlo uninformative variable elimination (MC-UVE)^29–33^, subwindow permutation analysis (SPA)^34,35^ and latent projective graph (LPG)^36,37^.

Previously, we used MC-UVE method to screen the NIR spectrum information of *W. cocos* ^38^. On this basis, in this study, four variable selection methods including CARS, MC-UVE, SPA and LPG were employed and compared for NIR variables selection. The common variables were selected from the variable selection results of the four methods. Then, the characteristic variables were screened based on the common variables for the establishment of Fisher function models in further identification of the origin of *W. cocos* from Yunnan, China. Additionally, the characteristic variables were also interpreted.

## Results and Discussion

### Stability of NIR

The NIR resulting.spc files were converted to.csv data files by the multivariate statistical analysis of SIMCA-P 11.0. The stability of 25 times parallel collections of a sample was considered by Hotelling T^2^. The results showed that the parallel spectrum acquisitions possessed satisfactory stability with coefficient 4.26 and 7.82 in the 95% and 99% levels in *W. cocos*, respectively. The results indicated that NIR was a reliable method for discriminant analysis.

### Principal Component Analysis

**Figure 1:**
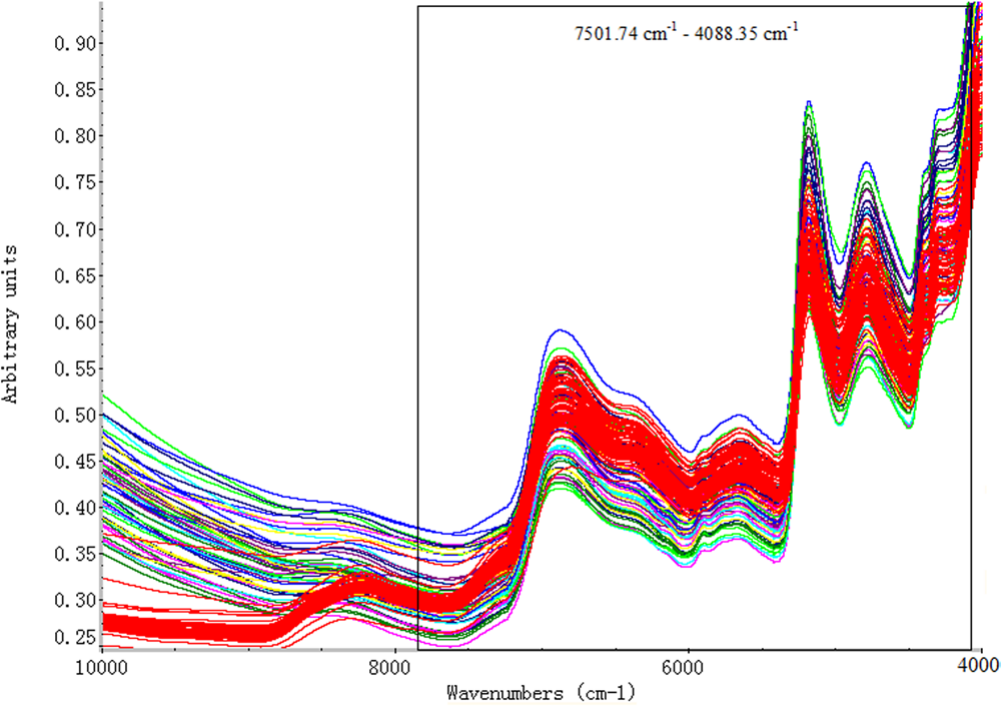
The original spectra of BFL and FLP. The red lines represent BFL samples, while the other colorized lines stand for FLP samples.

**Figure 2:**
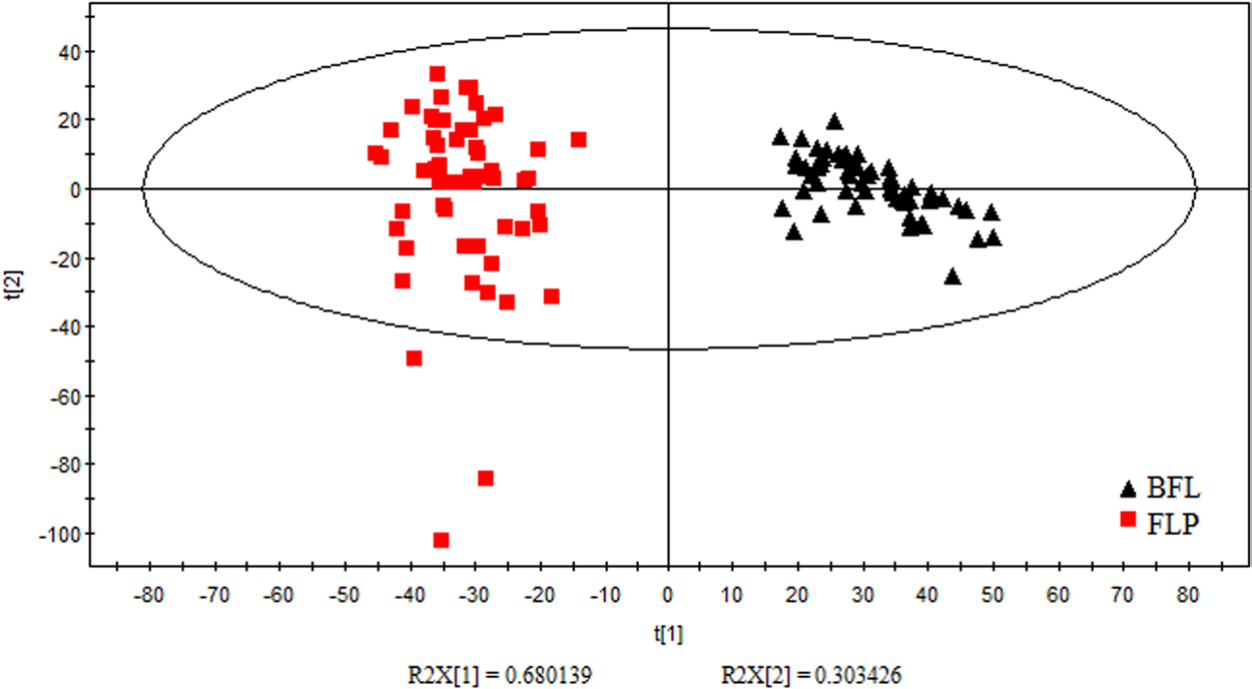
Principal component scores of BFL and FLP. The black triangles represent BFL samples, while the red squares correspond to FLP samples.

In order to remove the redundant information produced by hi gh-frequency line noise and retain the useful information in the low-frequency region, we applied the spectrum standard deviation (SDD) method to filter the original spectra by TQ 9.2^39^. The wave band 7501.74 cm^−1^ – 4088.35 cm^−1^ (886 wavelength points) was preliminary selected (as shown in Fig. 1). Then we analyzed *W. cocos* by principal component analysis (PCA). In Fig. 2, we could find that in the pattern space of PCA, BFL and FLP were completely separated. The result indicated the inner chemical compositions of the two parts were different. In view of this, we established the discriminant models of BFL and FLP separately.

We analyzed BFL and FLP by PCA, respectively. The results were shown in Supplementary Table S1. According to Kaiser Criterion, only factors with eigenvalues greater than or equal to one will be accepted as possible sources of variance in the data^40^. The first five factors that accounted for spectrum cumulative 97.858% of BFL and 97.203% of FLP were selected for the next analysis.

### Abnormal Samples Diagnosis

In the course of spectrum information (X) collection and index (Y) measurement, the data (X or Y) might deviate along with the abnormal fluctuation of instrument. The outlier samples could interfere with the discrimination model seriously. Through modular group iterative singular samples diagnosis method, the BFL and FLP were analyzed by Matlab R2010a analysis software. In order to establish steady discriminant model, the exceptional spectra including the number of samples 43 of BFL, 3, 33 and 35 of FLP were removed (see Supplementary Fig. S1).

### Classification of Training Set and Validation Set

According to K-S method^41,42^, the samples were divided into the training and validation sets of BFL and FLP by the proportion of 2:1, respectively. The training and validation sets of BFL contained 40 and 19 samples, and those of FLP had 39 and 18 samples, respectively. Each set included the samples of all the five regions. The training set was used for variable selection and modeling, and the independent validation set was used for validation of the model.

### Variable Selection based on CARS

The preliminary selected dataset 7501.74 cm^−1^–4088.35 cm^−1^ (886 wavelength points) was intended for investigating the ability for CARS to select key variables by eliminating the redundant information. One hundred replicate running of CARS was executed and the root mean square error of cross validation (RMSECV) values were recorded.

By 10-flod cross validation, the optimal number of PCA was five. The statistics of frequency of each selected wave number of spectrum was implemented. The number of Monte Carlo iterations was set to 50. In each iteration, 80% samples from the training sets were randomly chosen to build a PLS-DA model. The optimized number of variables was confirmed with the lowest RMSECV value. Only a small part of the wavelengths could be selected by CARS. According to the lowest RMSECV values, twenty key variables of FLP (RMSECV = 1.6202) were screened, and forty significant variables of BFL (RMSECV = 1.6767) were selected. Compared with preliminary selected variables (886 wavelength points), the optimized number of variables by CARS was reduced significantly (see Supplementary Fig. S2).

### Variable Selection based on MC-UVE

Five hundred replicate running of MC-UVE was executed and the RMSECV values were recorded. Ten-fold cross validation and five principal factors of PLS-DA model were used in this study to explore its prediction performance. Reliability index (RI), defined as the ratio of the mean to the standard deviation of this distribution, was used to assess the reliability of each variable. Based on this reliability, all variables were ranked. Then, these variables were sequentially added to build a PLS-DA model whose performance was assessed by cross validation. The RI corresponding to the variable whose addition results in the minimum RMSECV value was chosen as the threshold. The variables that were related with a RI lower than the threshold value could be removed^35^.

The analysis result showed the variables with the RI values greater than 2.5107 were selected using ten-fold cross validation for BFL, and 95 variables were selected when the minimum ten-fold RMSECV was 1.5601. For FLP, 35 variables with the RI values greater than 2.1589 were selected using ten-fold cross validation as the minimum ten-fold RMSECV was 1.5852 (see Supplementary Fig. S3).

### Variable Selection based on SPA

The three parameters of SPA were set to N = 1000 (N, the number of Monte Carlo Simulation), R = 0.8 (R, the ratio of samples to be selected in each Monte Carlo sampling), Q = 10 (Q, the number of variables to be sampled in each Monte Carlo Simulation). 10-flod cross validation and five number of PCA were used in this study to explore its prediction performance. The variable importance assessed by conditional synergetic score (COSS) value was calculated (COSS = − log_10_ (*P*)). RMSECV values were recorded, and the corresponding minimum RMSECV value was chosen as the optimized number of variables. The more significant a variable was, the higher the score it got. Particularly, the variables with COSS values greater than 2 were selected. As the minimum RMSECV value was 1.6235, 90 informative variables of BFL were selected for further analysis. For FLP, as the minimum RMSECV value was 1.6428, 30 informative variables were selected (see Supplementary Fig. S4).

### Variable Selection based on LPG

LPG^36^ was adopted in wavelength selection for NIR spectral analysis. The method calculated an LPG (score plot) by performing PCA on the NIR spectral data matrix (7501.74 cm^−1^–4088.35 cm^−1^), and then detected the non-collinear variables from the LPG. According to the results of PCA in Supplementary Table S1, the first two principal components were used for LPG. In the end, both BFL and FLP, 129 variables were selected by LPG (see Supplementary Figs S5 and S6).

### Evaluation of the Selected Variables

For further analysis the reliability of CARS, MU-UVE, SPA and LPG methods, PLS-DA models of BFL and FLP were established by SIMCA-P 11.0 software. The performance of models was assessed by determination coefficient (*R* ^2^), RMSECV and root mean square error of prediction (RMSEP). Generally, a good model should have high value of R^2^ and low value of RMSECV^43^. According to Galtier discriminant criterion, the ability of classification was assessed by prediction sets, and values of prediction and deviation (Y_pre_ and Y_dev_) were examined. When Y_pre_ > 0.5 and Y_dev_ < 0.5, the prediction samples belonged to a certain kind of training set; Y_pre_ < 0.5 and Y_dev_ < 0.5, the prediction samples did not belong to a certain kind of training set; Y_dev_ > 0.5 and 0.45 < Y_dev_ < 0.5, the prediction samples were suspicious, because they were very close to the threshold 0.5. The 0.45 and 0.55 limits have been chosen because they express 10% of error in the results^44,45^.

Tables 1 and 2 summarized the prediction results of the PLS-DA models performed on the extraction of NIR spectra by the different variables selection methods. Compared with the preliminary variables (7501.74 cm^−1^–4088.35 cm^−1^, 886 variables), through different variable selection methods (CARS, MC-UVE, SPA and LPG), the number of the selected variables were decreased. Simultaneously, the parameters for assessing the PLS-DA models were improved. The values of accuracy and *R* ^2^ increased, RMSECV and RMSEP reduced.

**Table 1:** Prediction results of PLS-DA models of BFL built by different variable selection methods.

Primary ID	886 spectral variables				40 spectral variables by CARS				95 spectral variables by MC-UVE				90 spectral variables by SPA				129 spectral variables by LPG			
	AC	CC	Y_pre_	Y_dev_	AC	CC	Y_pre_	Y_dev_	AC	CC	Y_pre_	Y_dev_	AC	CC	Y_pre_	Yd_ev_	AC	CC	Y_pre_	Y_dev_
BFL-01	1	1	0.836	0.116	1	1	1.001	0.001	1	1	0.654	0.245	1	1	1.023	0.016	1	1	0.805	0.138
BFL-05	1	1	1.629	0.445	1	1	0.774	0.160	1	1	0.644	0.252	1	1	1.23	0.163	1	1	0.548	0.320
BFL-20	1	1	1.536	0.379	1	1	0.834	0.117	1	1	0.541	0.324	1	1	0.805	0.138	1	1	0.749	0.178
BFL-34	1	1	1.618	0.437	1	1	0.719	0.199	1	1	0.621	0.268	1	1	1.205	0.145	1	1	0.762	0.168
BFL-40	1	1	0.835	0.117	1	1	1.168	0.119	1	1	0.711	0.204	1	1	1.577	0.408	1	1	0.822	0.126
BFL-48	1	SU	1.782	0.553	1	1	1.119	0.084	1	1	0.685	0.223	1	1	1.400	0.283	1	1	0.725	0.194
BFL-33	2	2	2.004	0.003	2	2	2.084	0.059	2	2	1.756	0.173	2	2	1.849	0.107	2	2	1.758	0.171
BFL-42	2	SU	2.635	0.450	2	2	1.554	0.315	2	2	1.539	0.326	2	2	1.87	0.092	2	2	1.682	0.225
BFL-49	2	2	1.928	0.051	2	2	1.593	0.288	2	2	2.192	0.136	2	2	1.963	0.026	2	2	1.920	0.057
BFL-54	2	2	1.845	0.110	2	2	1.967	0.023	2	2	1.963	0.026	2	2	1.72	0.198	2	2	1.821	0.126
BFL-55	2	2	1.751	0.176	2	2	1.942	0.041	2	2	1.882	0.083	2	2	2.034	0.024	2	2	1.716	0.201
BFL-12	3	3	2.802	0.140	3	3	3.019	0.013	3	3	2.562	0.310	3	3	2.88	0.085	3	3	2.575	0.300
BFL-15	4	4	3.844	0.110	4	4	4.593	0.419	4	4	3.744	0.181	4	4	3.876	0.088	4	4	3.582	0.296
BFL-37	4	4	3.948	0.037	4	4	3.712	0.204	4	4	3.720	0.198	4	4	3.9	0.071	4	4	3.807	0.137
BFL-47	4	4	3.893	0.076	4	4	3.873	0.090	4	4	3.861	0.098	4	4	3.657	0.243	4	4	3.817	0.129
BFL-04	5	SU	5.653	0.462	5	5	4.901	0.070	5	5	4.782	0.154	5	5	4.565	0.308	5	5	4.576	0.300
BFL-13	5	5	4.813	0.132	5	5	5.126	0.089	5	5	5.117	0.083	5	5	4.685	0.223	5	5	4.790	0.149
BFL-16	5	5	4.904	0.068	5	5	5.047	0.033	5	5	4.751	0.176	5	5	4.818	0.129	5	5	5.013	0.009
BFL-25	5	5	4.965	0.025	5	5	4.829	0.121	5	5	4.995	0.004	5	5	4.762	0.168	5	5	4.829	0.121
Accuracy (%)	84.21				100				100				100				100			
*R* ^2^	0.940				0.977				0.966				0.972				0.970			
RMSECV	0.290				0.181				0.219				0.197				0.208			
RMSEP	0.382				0.239				0.289				0.260				0.274			

Note: AC (Actual class), CC (Calculated class), Y_pre_ (Predicted value), Y_dev_ (Deviation), SU (Suspicious).

**Table 2:** Prediction results of PLS-DA models of FLP built by different variable selection methods.

Primary ID	886 spectral variables				20 spectral variables by CARS				35 spectral variables by MC-UVE				30 spectral variables by SPA				129 spectral variables by LPG			
	AC	CC	Y_pre_	Y_dev_	AC	CC	Y_pre_	Y_dev_	AC	CC	Y_pre_	Y_dev_	AC	CC	Y_pre_	Y_dev_	AC	CC	Y_pre_	Y_dev_
FLP-01	1	1	0.940	0.042	1	1	0.551	0.317	1	1	1.078	0.055	1	1	0.816	0.130	1	1	0.852	0.105
FLP-05	1	1	1.622	0.440	1	1	0.488	0.362	1	1	0.598	0.285	1	1	0.652	0.246	1	1	0.434	0.400
FLP-32	1	1	1.015	0.011	1	1	1.114	0.081	1	1	0.593	0.288	1	1	0.627	0.264	1	1	1.057	0.040
FLP-34	1	1	1.608	0.430	1	UN	0.380	0.438	1	1	1.290	0.205	1	1	1.172	0.122	1	1	0.974	0.018
FLP-40	1	1	0.799	0.142	1	1	1.133	0.094	1	1	0.609	0.277	1	1	1.013	0.009	1	1	0.906	0.066
FLP-50	1	1	0.810	0.134	1	1	1.138	0.098	1	1	0.866	0.095	1	1	1.436	0.308	1	1	0.870	0.092
FLP-59	1	1	0.617	0.271	1	1	0.971	0.021	1	1	0.579	0.298	1	1	1.321	0.227	1	1	0.647	0.249
FLP-30	2	2	1.895	0.074	2	2	1.474	0.372	2	2	2.465	0.329	2	2	1.802	0.140	2	2	1.737	0.186
FLP-46	2	2	2.681	0.482	2	2	1.502	0.352	2	2	1.701	0.212	2	2	2.47	0.332	2	2	1.604	0.280
FLP-49	2	2	2.587	0.415	2	2	2.053	0.037	2	2	1.509	0.347	2	2	1.866	0.095	2	2	1.521	0.339
FLP-54	2	2	1.855	0.102	2	2	1.566	0.307	2	2	1.663	0.238	2	2	1.657	0.243	2	2	1.962	0.027
FLP-08	3	3	2.989	0.008	3	3	3.368	0.260	3	3	3.039	0.028	3	3	3.469	0.332	3	3	2.899	0.072
FLP-26	4	4	3.616	0.271	4	4	3.900	0.071	4	4	4.463	0.327	4	4	3.779	0.156	4	4	3.828	0.121
FLP-45	4	4	4.412	0.291	4	4	4.350	0.247	4	4	3.723	0.196	4	SU	3.275	0.513	4	4	4.487	0.344
FLP-04	5	5	5.407	0.288	5	5	5.474	0.335	5	5	4.683	0.224	5	5	5.321	0.227	5	5	5.482	0.341
FLP-19	5	5	5.557	0.394	5	5	4.774	0.160	5	5	5.025	0.018	5	5	5.477	0.337	5	5	4.666	0.236
FLP-23	5	SU	5.727	0.514	5	5	4.785	0.152	5	5	5.335	0.237	5	5	4.589	0.291	5	5	4.760	0.170
FLP-25	5	5	4.777	0.158	5	5	4.599	0.284	5	5	4.789	0.149	5	5	4.720	0.198	5	5	4.809	0.135
Accuracy (%)	94.44				94.44				100				94.44				100			
*R* ^2^	0.932				0.950				0.958				0.949				0.964			
RMSECV	0.311				0.268				0.245				0.269				0.225			
RMSEP	0.410				0.353				0.323				0.354				0.296			

Note: AC (Actual Class), CC (Calculated Class), Y_pre_ (Predicted value), Y_dev_ (Deviation), UN (uncredited), SU (suspicious).

For BFL, the prediction accuracy values of the PLS-DA models performed on the extraction of NIR spectra by the four methods all reached 100%. The sequence of *R* ^2^ was CARS > SPA > LPG > MC-UVE, while they were in the exact opposite sequences for RMSECV and RMSEP as CARS < SPA < LPG < MC-UVE. All the four methods showed satisfactory prediction performance for BFL.

For FLP, the highest prediction accuracy values reached 100% in the PLS-DA models performed on the extraction of NIR spectra by MC-UVE and LPG methods, while 94.44% for CARS and SPA methods. The sequence of *R* ^2^ was LPG > MC-UVE > CARS > SPA. The values of RMSECV and RMSEP were in the opposite sequence LPG < MC-UVE < CARS < SPA. The results of MC-UVE and LPG were better than CARS and SPA for BFL.

The prediction results of the models were significant improved when conducting variable selection, and also the models were simplified by using only a small part of the variables. The results experimentally proved the necessity to perform variable selection before building a calibration model.

### Common Variables Analysis

Based on the variable selection results of the four methods, the variables which were selected more than twice were chosen as the common variables for the further analysis. Totally, there were 56 common variables of BFL and 21 common variables of FLP were chosen.

**Figure 3:**
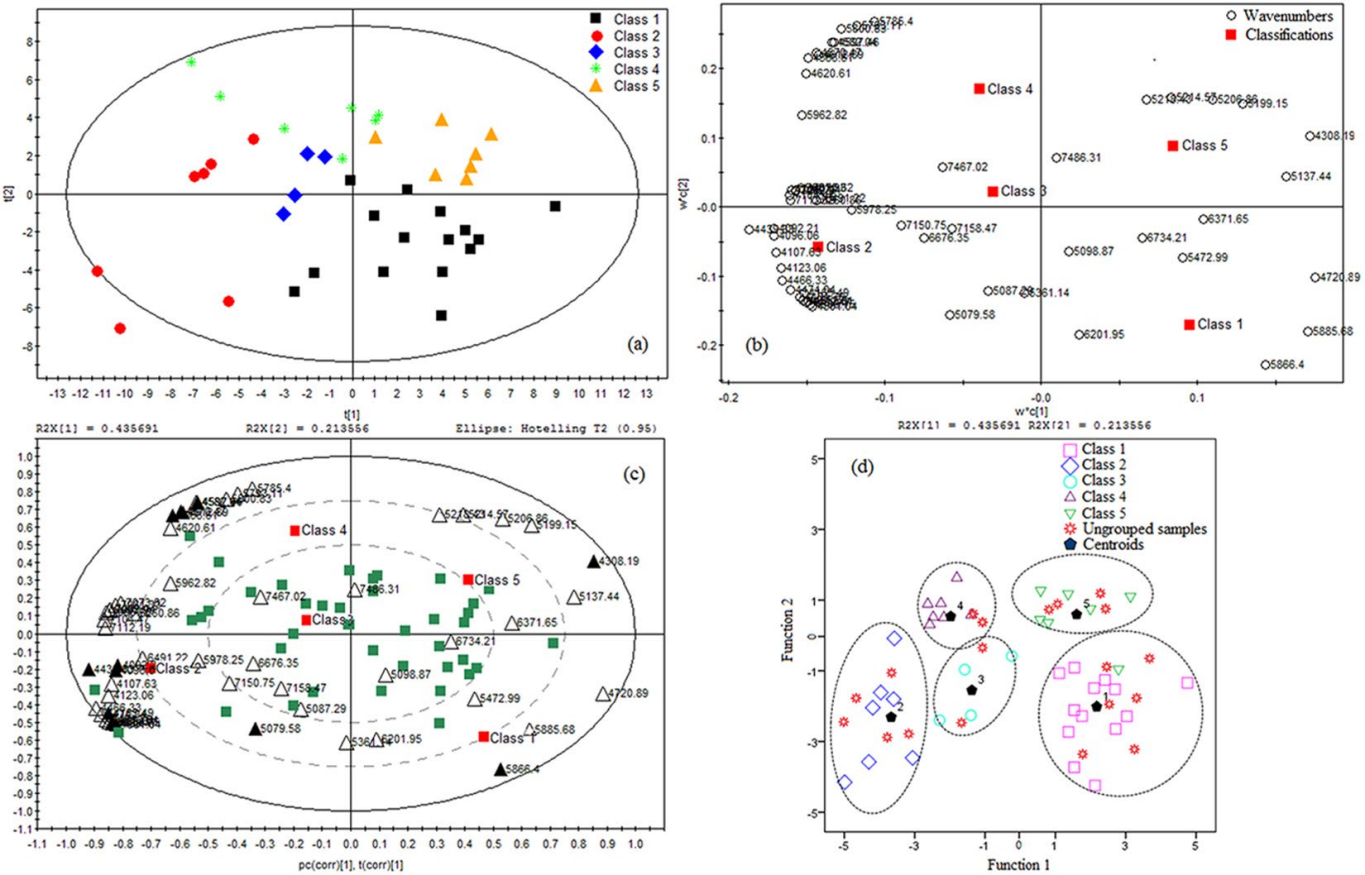
Chemometric analysis of common variables of BFL. (**a**) PLS-DA scores scatter plot. (**b**) PLS-DA loading scatter plot. (**c**) PLS-DA loadings biplot. (**d**) Fisher discriminant analysis scatter plot.

PLS-DA was performed based on the results of PCA of 56 common variables of BFL. From Fig. 3a, we found that the first two principal components cumulatively accounted for 64.9% of the variation. It was visible that BFL were separated into five groups. The loading scatter plot (Fig. 3b) displayed the contribution of each variable to the discrimination. The further the variable distance from the zero of the X-axis and the Y-axis, the more the variable contributes to the classification^46^. Through a visual analysis, the variables such as 4092.21, 4096.06, 4308.19 4439.33, 4597.46, 5079.58 and 5866.40 cm^−1^ were identified preliminarily. The biplot provided a better understanding about the relationships between samples and variables in one plot (Fig. 3c). The biplot displayed that the variables 5866.40 cm^−1^ was positively correlated with the samples in class 1 in the (+, −) quadrant. The variable 4597.46 cm^−1^ was positively correlated with the samples in class 2, 3 and 4 in the (−, +) quadrant, and negatively correlated with those in class 1 in the (+, −) quadrant. The variables 4092.21, 4096.06, 4439.33 and 5079.58 cm^−1^ were positively correlated with the samples in class 1, 2 and 3 in the (−, −) quadrant, and negatively correlated with those in class 5 in the (+, +) quadrant. The variable 4308.19 cm^−1^ was positively correlated with the samples in class 5 in the (+, +) quadrant. Those variables were the most important markers to separate BFL samples into the five classes.

**Figure 4:**
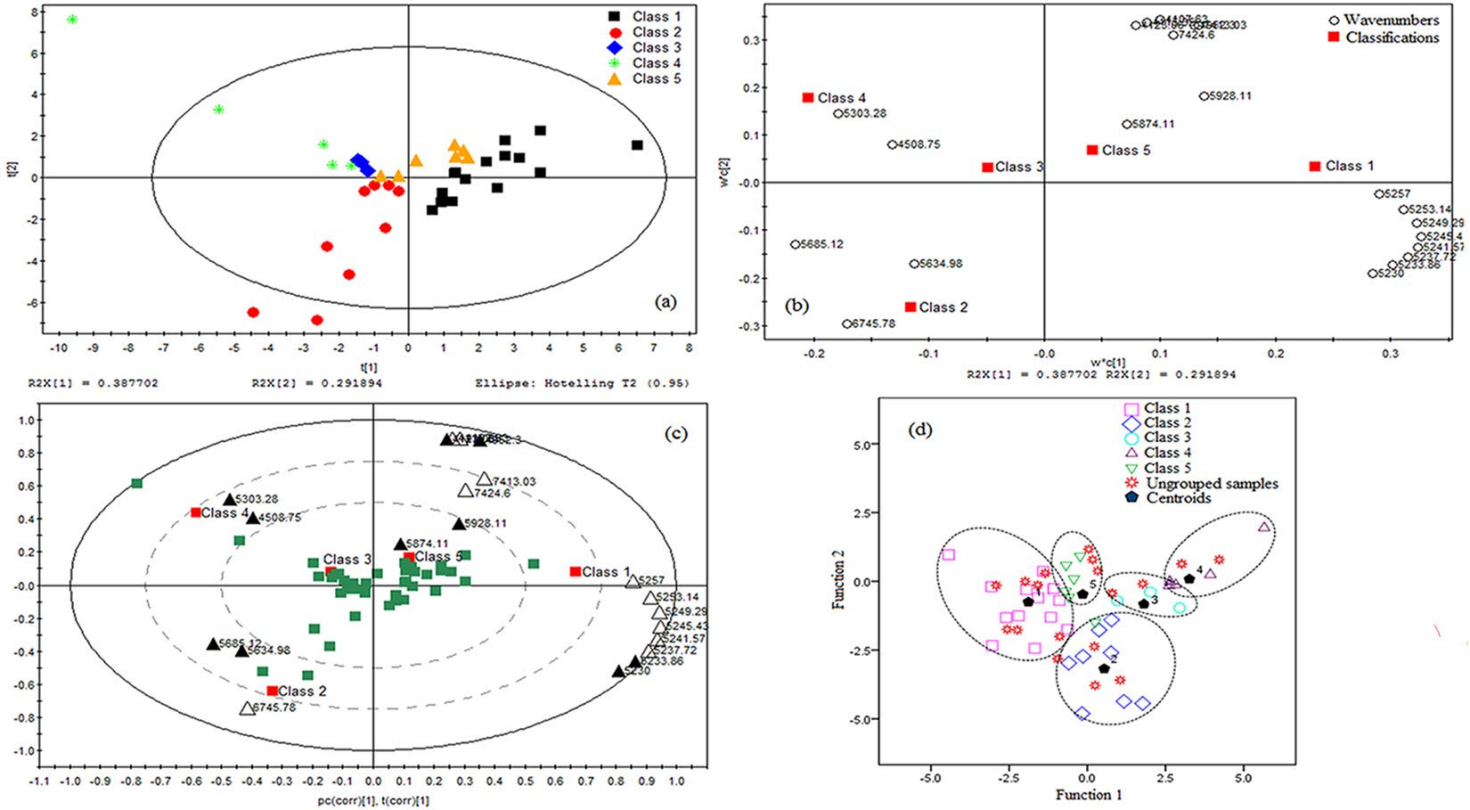
Chemometric analysis of common variables of FLP. (**a**) PLS-DA scores scatter plot. (**b**) PLS-DA loading scatter plot. (**c**) PLS-DA loadings biplot. (**d**) Fisher discriminant analysis scatter plot.

Simultaneously, PLS-DA was conducted for 21 common variables of FLP. In Fig. 4a, the first two principal components cumulatively accounted for 68.0% of the variation. The first principal component explained 38.8% of the total variance and the second principal component explained 29.2% of that. FLP samples were distinctly separated into five groups. Visually analyzed the loading scatter plot (Fig. 4b), we found the variables such as 4508.75, 4952.30, 5230.00, 5233.86, 5303.28, 5634.98, 5685.12, 5874.11 and 5928.11 cm^−1^ made a significant contribution to the discrimination. The biplot (Fig. 4c) showed that the variables 5230.00 and 5233.86 cm^−1^ were positively correlated with the samples in class 1 in the (+, −) quadrant. The variables 4508.75 and 5303.28 cm^−1^ were positively correlated with the samples in class 3 and 4 in the (−, +) quadrant, and negatively correlated with those in class 1 in the (+, −) quadrant. The variable 5634.98 and 5685.12 cm^−1^ were positively correlated with the samples in class 2 in the (−, −) quadrant, and negatively correlated with those in class 1 and 5 in the (+, +) quadrant. The variables 4952.30, 5874.11 and 5928.11 cm^−1^ were positively correlated with the samples in class 1 and 5 in the (+, +) quadrant. Those variables were the most important markers to separate FLP samples into the five classes.

### Establish of Discriminant Analysis Function

To identify and analyze the unknown samples, the Fisher discriminant function model was established. Through stepwise regression method, the common variables which made a greater contribution to classification were further screened. As a result, thirteen variables including 4092.21, 4096.06, 4165.49, 4308.19, 4439.33, 4485.61, 4501.04, 4566.61, 4570.47, 4597.46, 4612.89, 5079.58 and 5866.40 cm^−1^ were selected for BFL. Seven of them were identified in the above discussion of PLS-DA. Ten variables including 4123.06, 4508.75, 4952.30, 5230.00, 5233.86, 5303.28, 5634.98, 5685.12, 5874.11 and 5928.11 cm^−1^ were selected for FLP. Nine of them were recognized in the discussion of PLS-DA. The results of stepwise regression were in accordance with PLS-DA, which proved that those variables could be seen as the characteristic identification marks of *W. cocos*.

In the process of Fisher discriminant analysis, the thirteen variables of BFL and ten variables of FLP were used as discriminant variables respectively, and the different BFL and FLP samples were performed as the subjects of the study to establish Fisher discriminant functions. The function of BFL was shown as follow and the coefficients were in Table 3:

$$\begin{array}{c}\begin{array}{rcl}{\rm{Y}} & = & -{{\rm{A}}}_{0}+{{\rm{A}}}_{1}{{\rm{X}}}_{1}-{{\rm{A}}}_{2}{{\rm{X}}}_{2}-{{\rm{A}}}_{3}{{\rm{X}}}_{3}+{{\rm{A}}}_{4}{{\rm{X}}}_{4}+{{\rm{A}}}_{5}{{\rm{X}}}_{5}+{{\rm{A}}}_{6}{{\rm{X}}}_{6}-{{\rm{A}}}_{7}{{\rm{X}}}_{7}\\  &  & +{{\rm{A}}}_{8}{{\rm{X}}}_{8}-{{\rm{A}}}_{9}{{\rm{X}}}_{9}-{{\rm{A}}}_{10}{{\rm{X}}}_{10}+{{\rm{A}}}_{11}{{\rm{X}}}_{11}+{{\rm{A}}}_{12}{{\rm{X}}}_{12}+{{\rm{A}}}_{13}{{\rm{X}}}_{13}\end{array}\end{array}$$

where X_i_ was the corresponding variables, Y_i_ was the corresponding class.

**Table 3:** The coefficients of Fisher functions of BFL.

	Y_1_	Y_2_	Y_3_	Y_4_	Y_5_
A_0_	3698.54	4029.41	3761.75	3749.1	3788.01
A_1_	8.31E^+07^	8.18E^+07^	8.80E^+07^	7.77E^+07^	7.97E^+07^
A_2_	7.73E^+07^	7.58E^+07^	8.20E^+07^	7.10E^+07^	7.39E^+07^
A_3_	5.84E^+07^	6.18E^+07^	5.92E^+07^	6.04E^+07^	6.03E^+07^
A_4_	6.01E^+06^	3.62E^+06^	4.34E^+06^	5.36E^+06^	5.96E^+06^
A_5_	4.03E^+06^	5.67E^+06^	5.35E^+06^	5.74E^+06^	4.64E^+06^
A_6_	1.69E^+08^	1.77E^+08^	1.71E^+08^	1.71E^+08^	1.73E^+08^
A_7_	1.51E^+08^	1.59E^+08^	1.55E^+08^	1.53E^+08^	1.55E^+08^
A_8_	7.42 E^+08^	7.79E^+08^	7.84E^+08^	7.48E^+08^	7.56E^+08^
A_9_	4.35E^+08^	4.65E^+08^	4.81E^+08^	4.52E^+08^	4.54E^+08^
A_10_	5.83E^+08^	5.79E^+08^	5.64E^+08^	5.47E^+08^	5.73E^+08^
A_11_	4.48E^+08^	4.40E^+08^	4.33E^+08^	4.20E^+08^	4.44E^+08^
A_12_	6.64E^+06^	8.38E^+06^	8.07E^+06^	7.87E^+06^	6.05E^+06^
A_13_	2.73E^+07^	2.27E^+07^	2.36E^+07^	2.37E^+07^	2.77E^+07^

The function of FLP was shown as follow and the coefficients were in Table 4:

$${\rm{Y}}=-{{\rm{B}}}_{0}-{{\rm{B}}}_{1}{{\rm{T}}}_{1}+{{\rm{B}}}_{2}{{\rm{T}}}_{2}+{{\rm{B}}}_{3}{{\rm{T}}}_{3}+{{\rm{B}}}_{4}{{\rm{T}}}_{4}-{{\rm{B}}}_{5}{{\rm{T}}}_{5}+{{\rm{B}}}_{6}{{\rm{T}}}_{6}-{{\rm{B}}}_{7}{{\rm{T}}}_{7}+{{\rm{B}}}_{8}{{\rm{T}}}_{8}+{{\rm{B}}}_{9}{{\rm{T}}}_{9}-{{\rm{B}}}_{10}{{\rm{T}}}_{10}$$

where T_i_ was the corresponding variables, Y_i_ was the corresponding class.

**Table 4:** The coefficients of Fisher functions of FLP.

	Y_1_	Y_2_	Y_3_	Y_4_	Y_5_
B_0_	1075.23	1133.72	1171.49	1174.26	1126.33
B_1_	7.48E^+06^	7.47E^+06^	7.39E^+06^	7.23E^+06^	7.43E^+06^
B_2_	1.07E^+06^	6.71E^+05^	4.47E^+05^	3.29E^+07^	7.55E^+05^
B_3_	9.58E^+06^	9.37E^+06^	9.41E^+06^	9.30E^+06^	9.48E^+06^
B_4_	7.00E^+06^	8.03E^+06^	8.10E^+06^	8.57E^+06^	7.48E^+06^
B_5_	5.97E^+06^	7.05E^+06^	7.28E^+06^	7.98E^+06^	6.57E^+06^
B_6_	5.41E^+06^	5.90E^+06^	5.86E^+06^	5.86E^+06^	5.64E^+06^
B_7_	9.71E^+07^	9.82E^+07^	1.00E^+08^	9.89E^+07^	9.93E^+07^
B_8_	4.87E^+07^	4.91E^+07^	4.91E^+07^	4.98E^+07^	4.95E^+07^
B_9_	1.65E^+07^	1.68E^+07^	1.69E^+07^	1.63E^+07^	1.66E^+07^
B_10_	1.42E^+07^	1.53E^+07^	1.55E^+07^	1.47E^+07^	1.44E^+07^

The Fisher discriminant analysis results were shown in Figs 3d and 4d. The effect of discrimination model was evaluated by cross validation. As seen in the two figures, the ungrouped prediction samples located in different classes. The class of the ungrouped samples could be identified according to the distance from each sample to the centroids of all classes. The validation results were shown in Tables 5 and 6. The original grouped samples 97.50% for BFL and 97.43% for FLP were correctly classified. In the cross validation, the accuracy rates were 94.74% for BFL and 94.44% for FLP. In our previous study, the Fisher discriminant analysis functions built based on the wavelength selected only by the MC-UVE method, the original grouped samples 92.50% for BFL and 92.86% for FLP were correctly classified, and the accuracy rates were 80.95% for BFL and 83.33% for FLP in the cross validation^38^. The correct classification rates were significantly improved both in the original grouped samples and in the cross validation sets in this study. The validation results indicated that the Fisher discriminant function model established based on the characteristic variables selected simultaneously by the four methods CARS, MC-UVE, SPA and LPG could be seen as a reliable and effective method to discriminate BFL and FLP.

**Table 5:** The validation results of the Fisher discriminant analysis of BFL.

Validation	Statistics	Class	1	2	3	4	5	Total
Original^a^	Count	1	13	0	0	0	0	13
		2	0	7	0	0	0	7
		3	0	0	4	0	0	4
		4	0	0	0	6	0	6
		5	1	0	0	0	9	10
	Accuracy rate %		100	100	100	100	88.9	40
Cross validation^b^	Count	1	6	0	0	0	0	6
		2	0	5	0	0	0	5
		3	0	0	1	0	0	1
		4	0	0	1	2	0	3
		5	0	0	0	0	4	4
	Accuracy rate %		100	100	100	66.7	100	19

Note: ^a^97.50% % of original grouped cases correctly classified; ^b^Cross validation is done only for those cases in the analysis. In cross validation, each case is classified by the functions derived from all cases other than that case. 94.74% of the cross validation grouped cases correctly classified.

**Table 6:** The validation results of the Fisher discriminant analysis of FLP.

	Statistics	Class	1	2	3	4	5	Total
Original^a^	Count	1	13	0	0	0	0	13
		2	0	8	0	0	0	8
		3	0	0	3	0	0	3
		4	0	0	0	7	0	7
		5	1	0	0	0	7	8
	Accuracy rate %		100	100	100	100	87.5	39
Cross validation^b^	Count	1	6	0	0	0	0	6
		2	0	4	0	0	0	4
		3	0	0	2	0	0	2
		4	0	0	0	2	0	2
		5	1	0	0	0	3	4
	Accuracy rate %		100	100	100	100	75	18

Note: ^a^97.43% of original grouped cases correctly classified; ^b^Cross validation is done only for those cases in the analysis. In cross validation, each case is classified by the functions derived from all cases other than that case. 94.44% of the cross validation grouped cases correctly classified.

### Interpretation of the Characteristic Variables

In order to further understand the significance of these characteristic variables, we interpreted the spectra-structure of them. The wavelengths at 4092.21, 4,096.06, 4123.06, 4165.49, 4566.61 and 4570.47 cm^−1^ are related to the vibration of C-H aryl in benzene band. The absorption band at 4308.19 cm^−1^ is the combination of C-H stretch and C-H_2_ deformation in polysaccharides. The wavelength at 4439.33 cm^−1^ is the combination of O-H and C-O stretch in glucose. Band at 4485.61 is assigned as second overtones of the symmetric and asymmetric bending vibrations of the CH_2_ of the uncoupled vinyl group. Absorbance peaks at 4501.04 and 4508.75 cm^−1^ are the combination of asymmetric stretch of NH and NH_2_ rocking in urea (NH_2_-C=O-NH_2_). Absorbance peak at 4597.46 cm^−1^ is due to CONH_2_ as combination of amide B and amide II modes. The wavelength at 4612.89 cm^−1^ is assigned to CONH_2_ specifically due to the α-helix peptide structure. The absorption band at 5079.58 cm^−1^ is the combination of N-H stretching vibration and N-H bending in aromatic amine. Absorbance peak at 5866.40 cm^−1^ corresponds to C-H first overtone stretch vibration mode in CH_3_. The absorption band at 4952.30 cm^−1^ is due to a combination of the OH stretch and CH bending. The wavelengths at 5230.00, 5233.86 and 5303.28 cm^−1^ are the hydroxyl bands. The peaks at 5634.98 and 5685.12 cm^−1^ are related to C-H in methylene. The band at 5874.11 cm^−1^ is assigned to C-H in methyl, while at 5928.11 cm^−1^ is C-H in methyl with OH associated^47^. According to the absorption peaks, we could speculate that the chemical compositions of BFL and FLP were different, which provided theoretical basis in the spectrum level for the traditional usage of cutis (FLP) and the inner part (BFL) of the sclerotia of *W. cocos* separately.

## Conclusions

In this work, we first systematically collected the near-infrared spectrum of cutis (FLP) and the inner part (BFL) of the sclerotia of *W. cocos* from different regions in Yunnan, China. Interestingly, we found that there were obvious differences between FLP and BFL in the pattern space of PCA. Based on this, we established discriminant models for FLP and BFL separately. Through four variable selection methods CARS, MC-UVE, SPA and LPG, the common variables were selected. Furthermore, the characteristic variables were screened to build Fisher discriminant function models, and the validation results showed the models were reliable and effective. The variable selection method used in NIR spectrum provided a new thought for the origin identification of traditional Chinese medicines. The spectrum difference between the cutis (FLP) and the inner part (BFL) of the sclerotia of *W. cocos* provided theoretical basis in the spectrum level for the traditional usage of FLP and BFL separately.

## Methods

### Materials

Sixty *W. cocos* samples from five different areas of Yunnan Province in China were collected during July to August in 2015: the central Yunnan (19), western Yunnan (12), northwestern Yunnan (5), southwestern Yunnan (10) and southeastern Yunnan (14). They were identified and authenticated by Professor H. Jin, Yunnan Academy of Agricultural Sciences. The specimens were preserved in the Institute of Medicinal Plants, Yunnan Academy of Agricultural Sciences. The samples were separated into FLP and BFL. After drying at room temperature, samples were ground to fine powder and stored in the zip lock bags for further analysis. The detailed sample information is listed in Supplementary Table S2.

### Instruments

Antaris II Fourier Transform Near Infrared Spectroscopy (Thermo Fisher Scientific INC., USA) was attached with diffuse reflection module. The spectrum collecting software Result^TM^ 2.1 and the analysis software TQ 9.2 included in the instrument were employed. Traditional Chinese medicine grinder DFT-100 (Zhejiang wenling Linda machinery co., LTD) was applied. Stainless steel sieve tray 80 mesh (Tai’an of Chinese and western, Beijing) was used. The multivariate data analysis softwares were SIMCA-P 11.0 (Umetrics, Umea, Sweden), SPSS 19.0 (SPSS Inc., Chicago, USA) and MATLAB R2010a, and the code was derived from http://www.mathworks.cn/.

### Spectra Collection

The powder (20.0 g) was weighed before it was sufficiently mixed, then transferred to the sample cup of NIR and compressed. The parameters of collection were scanning (64 times), resolution (4 cm^−1^), scanning range (10000 cm^−1^–4000 cm^−1^) and parallel collection (3 times). The NIR spectra of *W. cocos* were preprocessed with Norris, mean centering, standardization, and second derivative successively by software TQ 9.2. Through optimizing, the range 7501.74–4088.35 cm^−1^ was selected according to the spectrum standard deviation. The higher the spectra standard deviation was, the greater a contribution made to classification.

## Electronic supplementary material

Supplementary Information
