# Supplementary material for: Application of variable selection in the origin discrimination of Wolfiporia cocos (F.A. Wolf) Ryvarden & Gilb. based on near infrared spectroscopy
Source: Sci Rep. 2018 Jan 8;8:89. doi: 10.1038/s41598-017-18458-9 (PMC5758700; doi:10.1038/s41598-017-18458-9)
Supplement: Supplementary file 1 — Supplementary Information [file 41598_2017_18458_MOESM1_ESM.pdf]

Application of variable selection in the origin discrimination of *Wolfiporia cocos* (F.A. Wolf) Ryvarden & Gilb. based on near infrared spectroscopy

Tianjun Yuan[1, 2], Yanli Zhao[1], Ji Zhang[1] & Yuanzhong Wang[1]

[1] Institute of Medicinal Plants, Yunnan Academy of Agricultural Sciences, Kunming 650200, China. [2] Yunnan Comtestor CO., LTD., Kunming 650106, China. Correspondence should be addressed to Y.Z. Wang (e-mail:boletus@126.com)

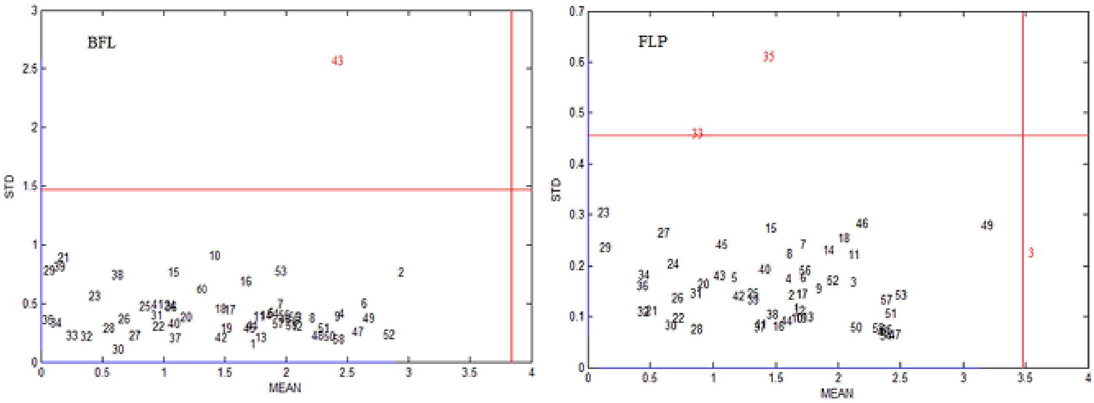

Supplementary Figure S1 Singular sample diagnosis. The number of samples 43 of BFL, 3, 33 and 35 of FLP were diagnosed as singular samples.

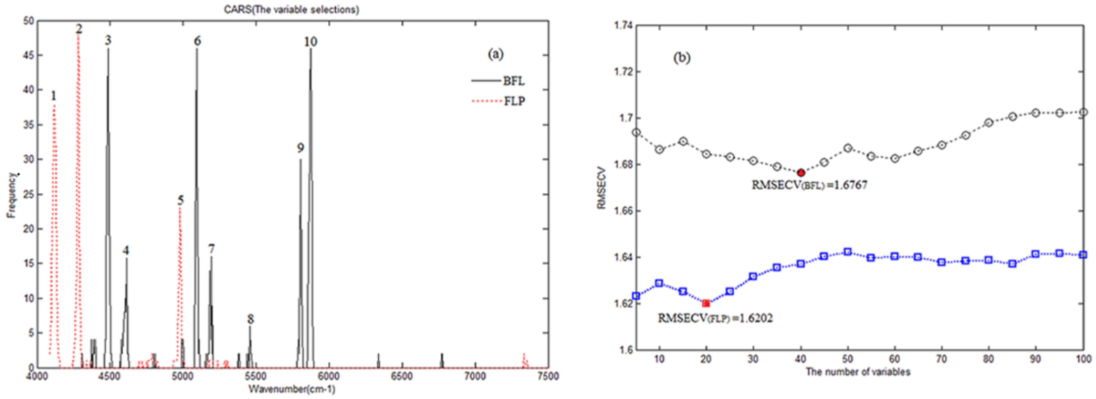

Supplementary Figure S2 The selected frequency (a) and number (b) of variables of BFL and FLP by CARS. The selected wavelengths were mainly distributed in three regions marked by 1, 2 and 5 for FLP, and seven regions denoted by 3, 4, 6, 7, 8, 9 and 10 for BFL, respectively.

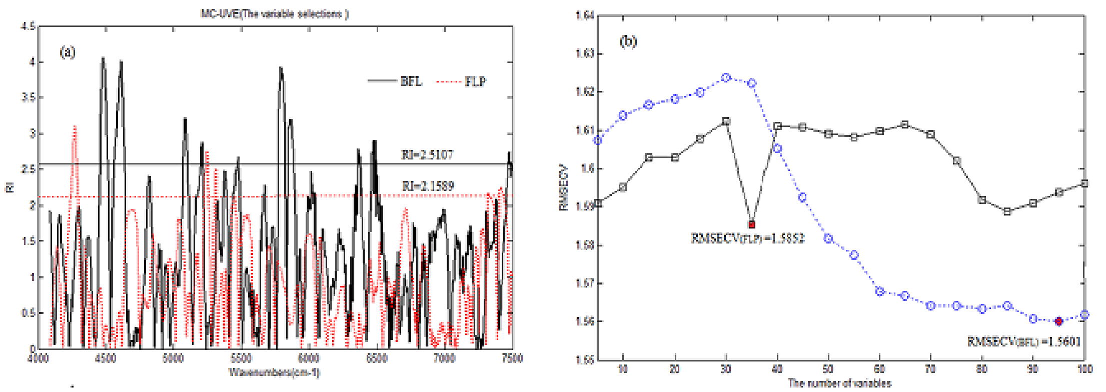

Supplementary Figure S3 RI threshold (a) and selected number (b) of variables of BFL and FLP by MC-UVE. The variables with the RI values greater than the thresholds were selected.

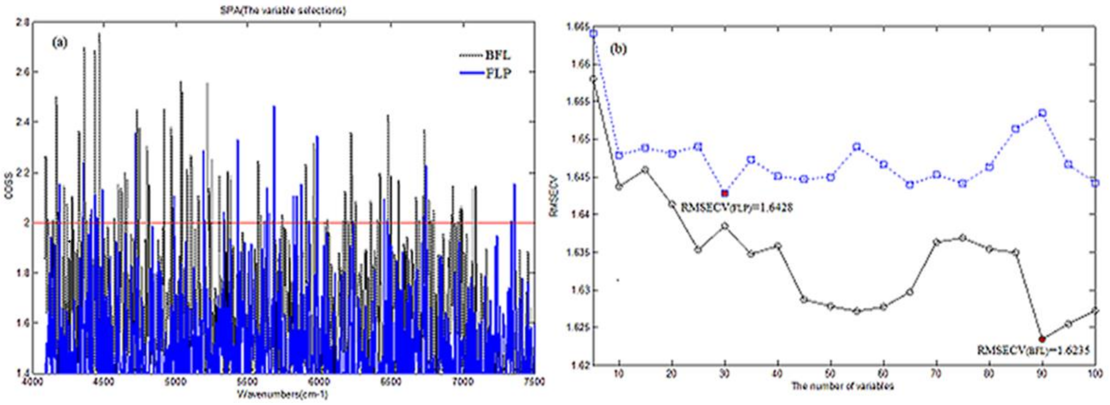

Supplementary Figure S4 COSS values (a) and selected number (b) of variables of BFL and FLP by SPA. The variables with COSS values greater than 2 were selected.

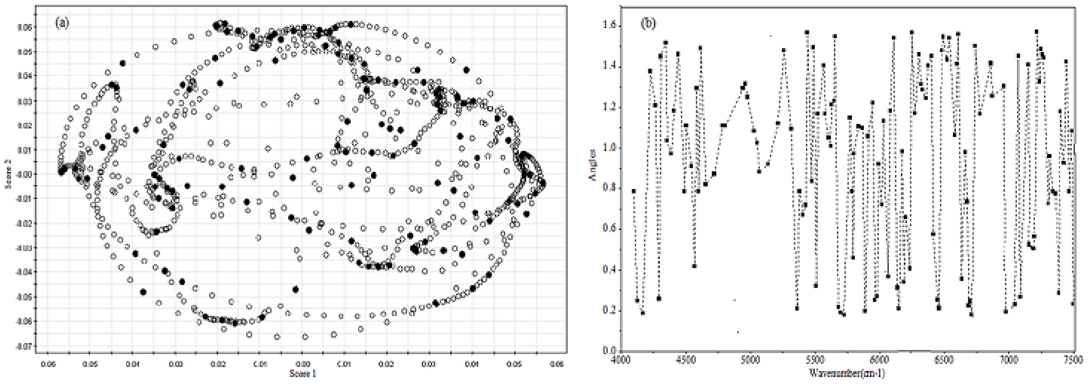

Supplementary Fig. S5 LPG (a) and the selected variables (b) of BFL. Fig. S5a showed the LPG (score 1 vs. score 2) of BFL, in which the solid dots were the variables at the inflections. Fig. S5b demonstrated the sites of the selected variables in the actual spectrum.

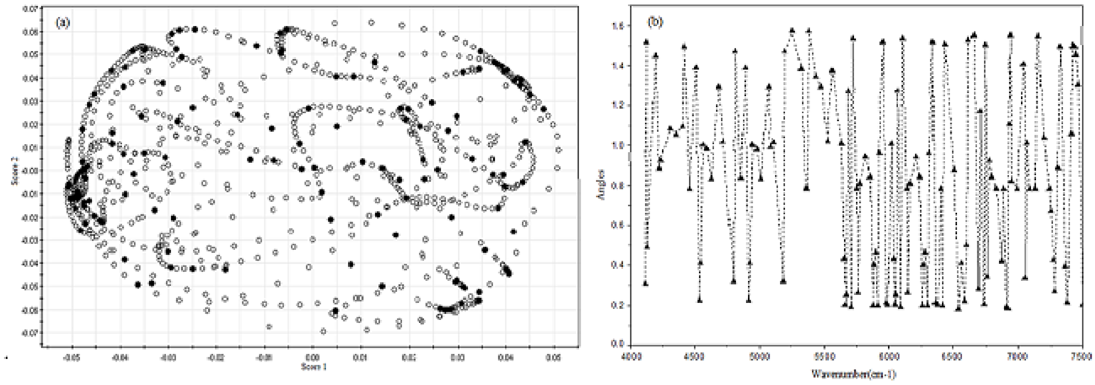

Supplementary Fig. S6 LPG (a) and the selected variables (b) of FLP. Fig. S6a showed the LPG (score 1 vs. score 2) of BFL, in which the solid dots were the variables at the inflections. Fig. S6b demonstrated the sites of the selected variables in the actual spectrum.

Supplementary Table S1 Selection of the number of principal component

Component	Initial eigenvalues			Initial eigenvalues		
	Total	% of variance of BFL	Cumulative % of BFL	Total	% of variance of FLP	Cumulative % of FLP
1	13.22	65.488	65.488	20.281	59.047	59.047
2	3.368	16.69	82.178	7.724	22.487	81.534
3	1.817	8.999	91.177	2.637	7.678	89.212
4	1.149	5.688	96.865	1.644	4.787	93.999
5	1.050	0.993	97.858	1.101	3.204	97.203

Supplementary Table S2 Detail information of *Wolfiporia cocos* samples from Yunnan Province.

Number	Collection sites	Classification	Number	Collection sites	Classification
FL-01	Yuan jiang, Yuxi	1	FL-31	Eshan, Yuxi	1
FL-02	Yuanjiang, Yuxi	1	FL-32	Eshan, Yuxi	1
FL-03	Jianshui, Honghe	5	FL-33	Nanhua, Chuxiong	2
FL-04	Luxi, Honghe	5	FL-34	Xinping, Yuxi	1
FL-05	Xinping, Yuxi	1	FL-35	Xincun, Chuxiong	2
FL-06	Shangri-La, Diqing	3	FL-36	Xinping, Yuxi	1
FL-07	Shangri-La, Diqing	3	FL-37	Zhenyuan, Pu’er	4
FL-08	Shangri-La, Diqing	3	FL-38	Mojiang, Pu’er	4
FL-09	Shiping, Honghe	5	FL-39	Xinping, Yuxi	1
FL-10	Zhenyuan, Pu’er	4	FL-40	Luquan, Kunming	1
FL-11	Tengchong, Baoshan	2	FL-41	Jingdong, Pu’er	4
FL-12	Ninglang, Lijiang	3	FL-42	Daguokou, Chuxiong	2
FL-13	Mile, Honghe	5	FL-43	Jingdong, Pu’er	4
FL-14	Shiping, Honghe	5	FL-44	Xinping, Yuxi	1
FL-15	Zhenyuan, Pu’er	4	FL-45	Zhenyuan, Pu’er	4
FL-16	Shiping, Honghe	5	FL-46	Shuangbai, Chuxiong	2
FL-17	Shiping, Honghe	5	FL-47	Jingdong, Pu’er	4
FL-18	Jianshui, Honghe	5	FL-48	Eshan, Yuxi	1
FL-19	Shiping, Honghe	5	FL-49	Shuzuo, Chuxiong	2
FL-20	Eshan, Yuxi	1	FL-50	Eshan, Yuxi	1

Number	Collection sites	Classification	Number	Collection sites	Classification
FL-21	Shiping, Honghe	5	FL-51	Nanhua, Chuxiong	2
FL-22	Shiping, Honghe	5	FL-52	Shuangbai, Chuxiong	2
FL-23	Kaiyuan, Honghe	5	FL-53	Xinping, Yuxi	1
FL-24	Jianshui, Honghe	5	FL-54	Shuangbai, Chuxiong	2
FL-25	Shiping, Honghe	5	FL-55	Dadiji, Chuxiong	2
FL-26	Jinggu, Pu’er	4	FL-56	Zhenyuan, Pu’er	4
FL-27	Eshan, Yuxi	1	FL-57	Xinping, Yuxi	1
FL-28	Xinping, Yuxi	1	FL-58	Xinping, Yuxi	1
FL-29	Xishelu, Chuxiong	2	FL-59	Xinping, Yuxi	1
FL-30	Bajiao, Chuxiong	2	FL-60	Weixi, Diqing	3

Note: The classification “1” represents the Central of Yunnan; “2” represents the western Yunnan; “3” represents the northwestern Yunnan; “4” represents the southwestern Yunnan; “5” represents the southeastern Yunnan.
